# Underwater Endoscopic Mucosal Resection as a Salvage Strategy for a Non‐Lifting Early Sigmoid Colon Carcinoma After Failed Conventional Endoscopic Mucosal Resection: A Case Report

**DOI:** 10.1002/deo2.70225

**Published:** 2025-10-22

**Authors:** Hiroshi Sawaguchi, Takuma Okamura, Yugo Iwaya, Hiroyoshi Ota, Tadanobu Nagaya

**Affiliations:** ^1^ Department of Medicine Division of Gastroenterology and Hepatology Shinshu University School of Medicine Nagano Japan; ^2^ Department of Laboratory Medicine Shinshu University School of Medicine Nagano Japan; ^3^ Department of Clinical Laboratory Sciences Shinshu University School of Medicine Nagano Japan

**Keywords:** colorectal carcinoma, endoscopic mucosal resection, non‐lifting sign, salvage therapy, underwater EMR

## Abstract

Endoscopic mucosal resection (EMR) has been widely adopted as an endoscopic treatment for colorectal tumors. However, in non‐lifting lesions, EMR often becomes technically challenging, leading to piecemeal resection or residual tumor. Recently, underwater EMR (UEMR) has been developed as a novel technique that allows mucosal and submucosal layers to float under water, facilitating snare resection without submucosal injection. UEMR has been reported to improve en bloc resection rates and shorten procedure time compared with conventional EMR, and its usefulness has been demonstrated in non‐lifting and residual lesions. We encountered a case in which a 78‐year‐old woman had a small IIa+IIc‐type lesion of the sigmoid colon that could not be completely removed by EMR at a previous hospital due to non‐lifting and snare slippage, resulting in only partial resection. She was subsequently referred to our hospital for further treatment. At our hospital, UEMR was successfully performed, achieving en bloc resection. Histopathological examination revealed well‐differentiated tubular adenocarcinoma with 560 µm submucosal invasion, negative resection margins, and no lymphovascular invasion, thus fulfilling the criteria for curative resection. This case highlights the illustrative and educational significance of applying UEMR, rather than endoscopic submucosal dissection, to achieve a safe and time‐efficient curative resection for a small non‐lifting colorectal carcinoma. UEMR may represent a potential salvage option in selected EMR‐difficult cases, although further accumulation of cases is warranted to clarify its role.

## 1 Introduction

Endoscopic mucosal resection (EMR) has become a standard technique for the endoscopic treatment of colorectal neoplasms. However, in cases with submucosal fibrosis or tumor invasion, sufficient mucosal elevation by submucosal injection cannot be achieved, resulting in instability of the snare and technical difficulty. Such situations, characterized by the “non‐lifting sign,” are associated with increased risks of piecemeal resection and residual tumor, ultimately leading to poorer treatment outcomes [1].

For non‐lifting lesions, endoscopic submucosal dissection (ESD) is widely applied with the aim of curative resection. Nevertheless, ESD requires a longer procedure time and carries a higher risk of complications, such as perforation [1, 2]. In contrast, underwater EMR (UEMR), which has recently attracted attention, involves filling the intestinal lumen with water to allow the mucosal and submucosal layers to float naturally. This technique enables snare resection without the need for submucosal injection. UEMR has been reported to improve en bloc resection rates and shorten procedure time compared with conventional EMR, and its efficacy has been particularly demonstrated in non‐lifting, residual, and recurrent lesions [3].

This case involved a small IIa+IIc‐type carcinoma of the sigmoid colon. At the referring hospital, EMR resulted in only partial resection due to non‐lifting; however, en bloc curative resection was successfully achieved using UEMR. Through this report, we discuss the clinical utility and significance of UEMR in the management of technically challenging colorectal cancers.

## 2 Case Report

**FIGURE 1:**
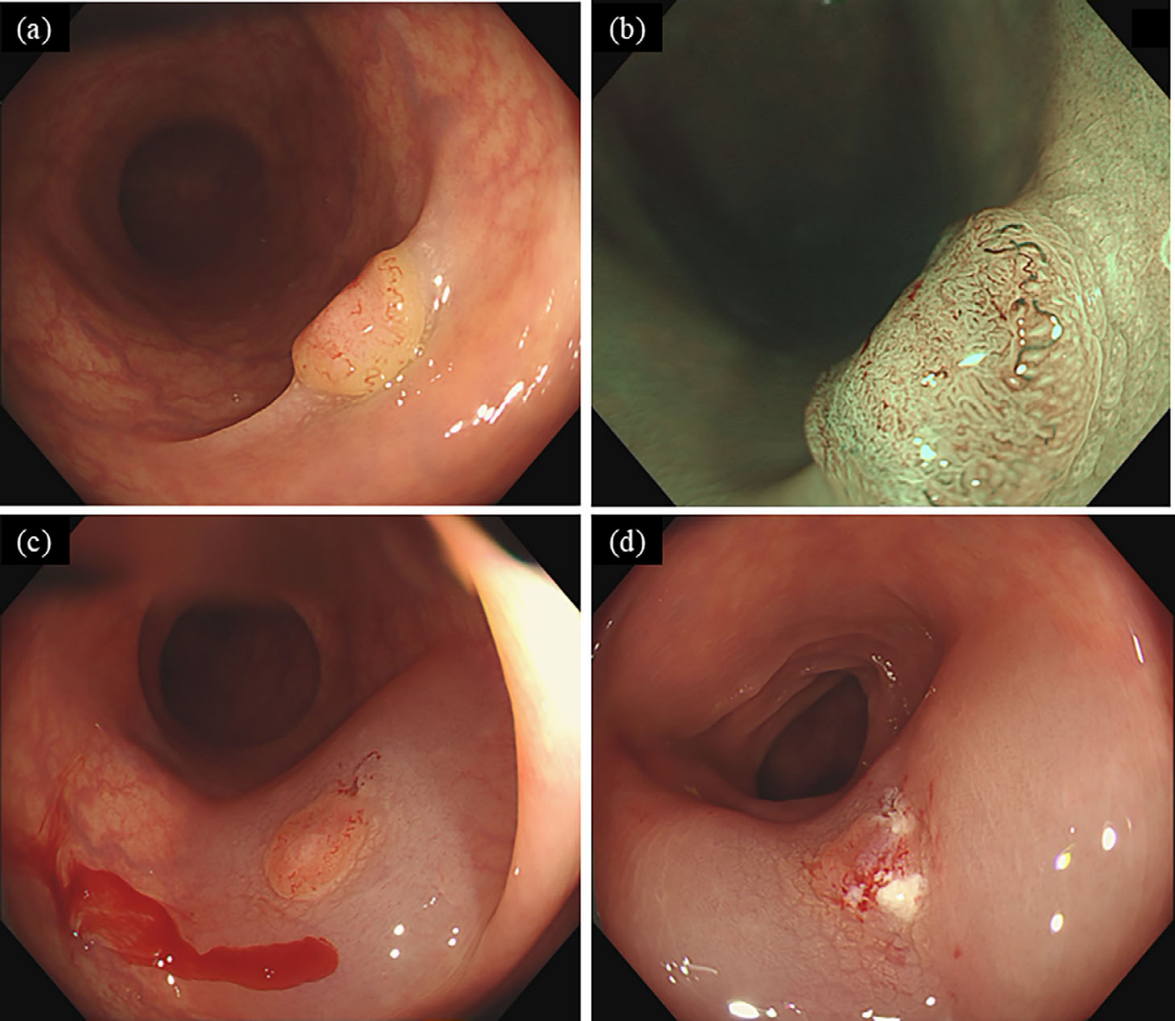
Endoscopic findings at the time of endoscopic mucosal resection (EMR) showed a IIa+IIc‐type lesion (a, b), with features suggestive of a de novo carcinoma. Submucosal injection was performed, but the lesion exhibited a positive non‐lifting sign (c). EMR resulted in only partial resection of the lesion surface (d).

The patient was a 78‐year‐old woman with no significant past medical history. During a routine health check‐up in 2025, a fecal occult blood test was positive, and she underwent a colonoscopy at a referring hospital. Colonoscopy revealed an approximately 7‐mm IIa+IIc‐type lesion in the sigmoid colon (Figure 1a,b), and EMR was attempted. However, submucosal injection failed to achieve adequate lifting of the central portion of the lesion (Figure 1c), and the snare slipped during resection, resulting in only partial removal of the surface (Figure 1d). Magnifying endoscopic findings (JNET Type 2B) were suggestive of carcinoma, and histopathological evaluation of the partially resected specimen confirmed adenocarcinoma. Therefore, the patient was referred to our hospital for further treatment.

**FIGURE 2:**
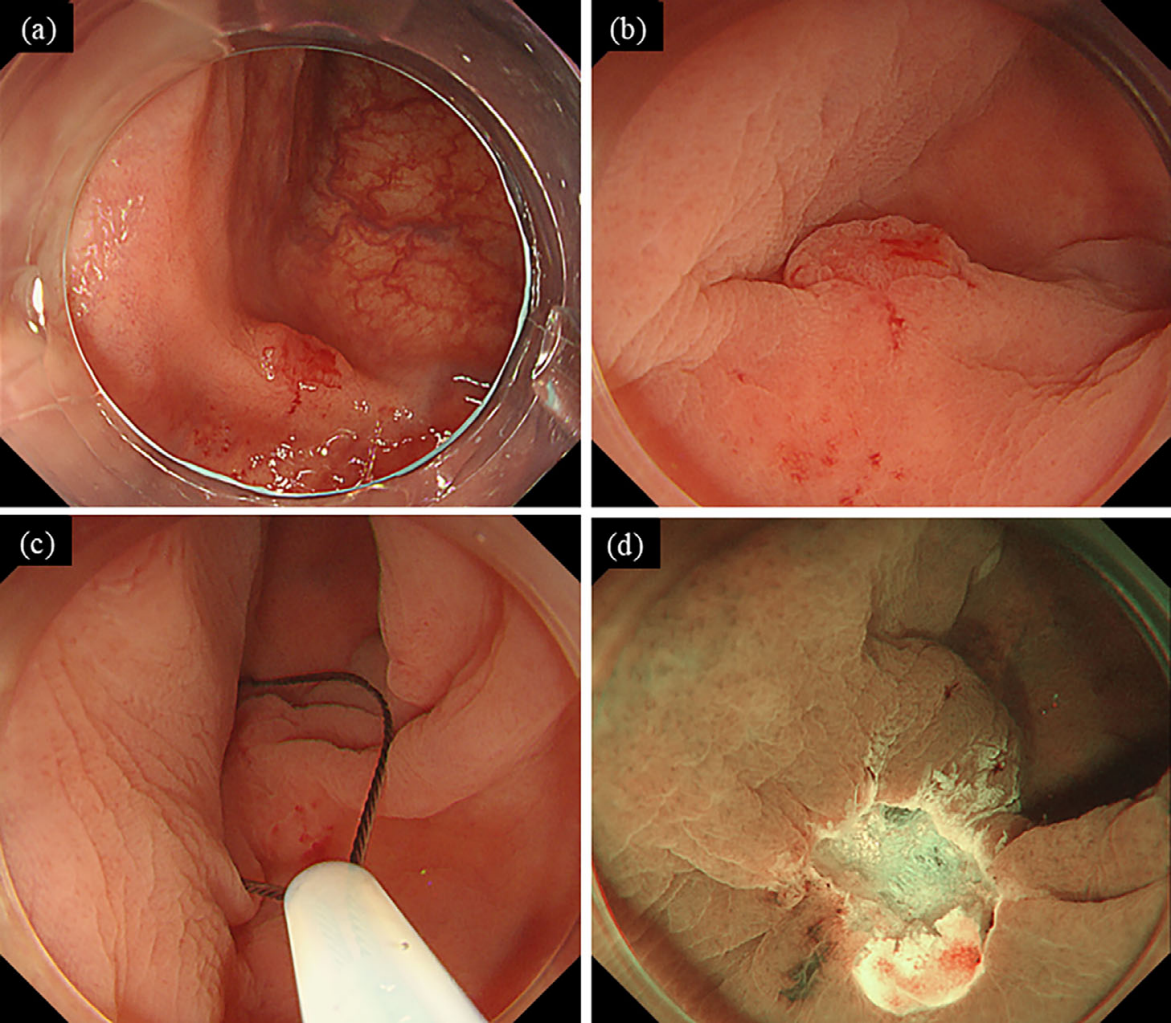
Endoscopic findings during underwater endoscopic mucosal resection (UEMR). On the anal side of the lesion, partial scarring with mucosal traction was observed (a). The lesion was visualized under water immersion (b), and UEMR was performed using a 10‐mm snare (c, d).

At our hospital, colonoscopy again revealed a 5–6‐mm IIa+IIc‐type lesion in the sigmoid colon. Due to the previous partial resection, the lesion appeared slightly smaller, with scarring and mucosal traction noted around its margins (Figure 2a,b). Submucosal invasion of the carcinoma or procedure‐related fibrosis was suspected, and resection by ESD or hybrid ESD was considered. However, given the small size of the lesion, UEMR was ultimately selected.

UEMR was performed using a 10‐mm snare (Captivator II; Boston Scientific, Marlborough, MA, USA) and an ESG‐100 electrosurgical generator (Olympus Medical Systems, Tokyo, Japan) under pulse‐cut slow mode at a power setting of 20 W (Figure 2c). During UEMR, the intestinal lumen was carefully decompressed to maintain collapse and a completely water‐immersed condition. A PCF‐H290TI colonoscope (Olympus Medical Systems, Tokyo, Japan) was used. When capturing the lesion, the snare was advanced under water with downward angulation applied to the scope tip, and deliberate pressure was exerted to ensure secure grasping of the lesion before resection. These steps, while consistent with standard UEMR, were particularly emphasized to overcome the technical challenges associated with fibrosis and scarring in this recurrent setting.

**FIGURE 3:**
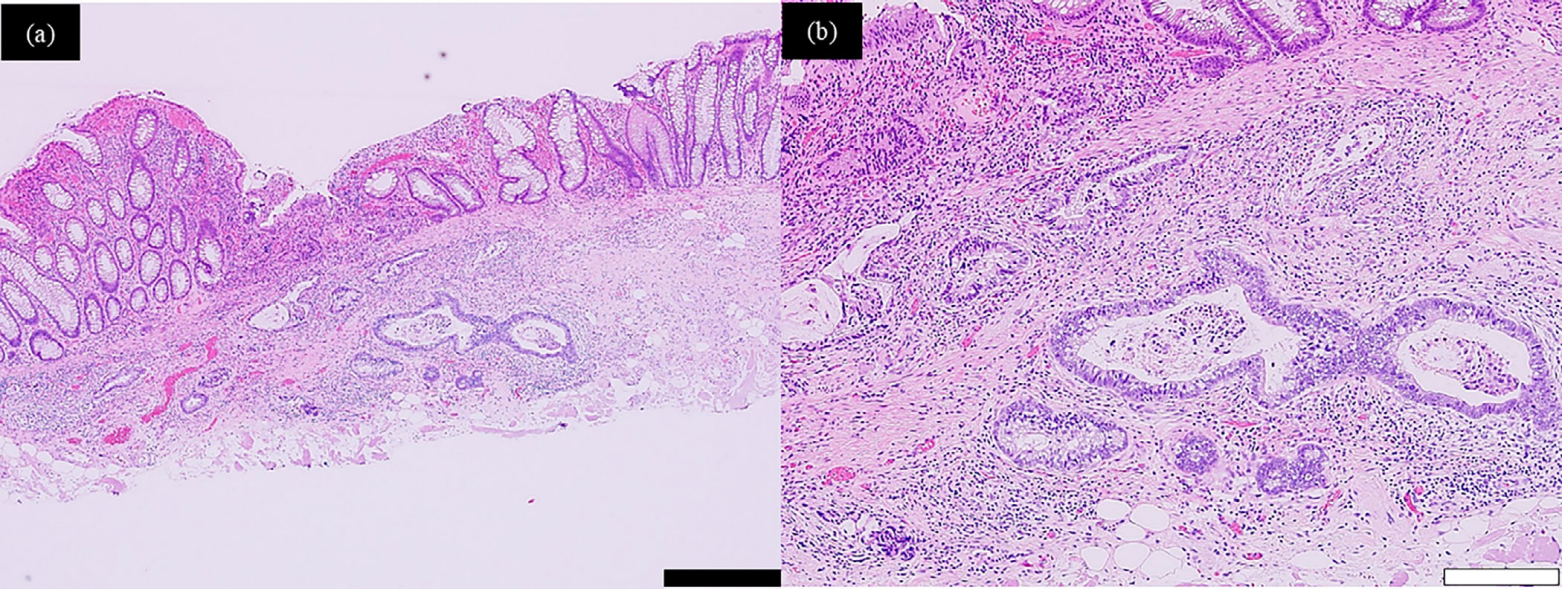
Histopathological findings of the resected specimen. Hematoxylin–eosin staining (a, b) confirmed adenocarcinoma infiltrating into the submucosal layer. Thickening of the muscularis mucosae and regenerative changes of the mucosal epithelium were also observed, consistent with changes after partial resection. The black scale bar indicates 500 µm, and the white scale bar indicates 200 µm.

The lesion was successfully resected en bloc, with negative lateral and deep margins (Figure 2d). Histopathological analysis revealed a well‐differentiated tubular adenocarcinoma (tub1) with 560 µm submucosal invasion, no lymphovascular invasion, and negative lateral and vertical margins, consistent with curative resection [2] (Figure 3a,b).

A follow‐up colonoscopy performed three months after treatment showed no evidence of residual or recurrent disease.

## 3 Discussion

This case involved a 7‐mm IIa+IIc‐type early carcinoma of the sigmoid colon that demonstrated a non‐lifting sign during EMR at the referring hospital, resulting in incomplete resection. At our institution, en bloc curative resection was successfully achieved using UEMR. The shallow submucosal invasion likely accounted for the inadequate elevation and technical failure of the initial EMR.

UEMR is performed by complete water immersion and luminal deflation, which reduces wall tension and allows the mucosal and submucosal layers to float. This facilitates snare capture and resection without submucosal injection. Since its first description, several studies have confirmed its usefulness in fibrotic, residual, recurrent, and non‐lifting lesions [4]. Snare deployment underwater increases the mobility of the lesion margins and enables selective capture without including the muscularis propria, providing clear mechanical advantages.

In comparative studies of UEMR versus conventional EMR (CEMR), a multicenter randomized controlled trial (RCT) of 10–20 mm lesions demonstrated that UEMR achieved significantly higher R0 and en bloc resection rates than CEMR (R0: 69% vs. 50%, en bloc: 89% vs. 75%) [3]. Similarly, an RCT of 20–40 mm lesions showed that UEMR achieved higher R0 and en bloc rates, fewer pieces, and shorter procedure times, with overall recurrence comparable [5]. A meta‐analysis also suggested that UEMR and CEMR are comparable in en bloc resection rates, R0 resection rates, procedure times, and complication profiles [6]. Additionally, for salvage treatment of incompletely resected or residual lesions, UEMR has been reported to achieve higher en bloc and complete resection rates, as well as lower argon plasma coagulation use and recurrence rates compared with CEMR [4].

When compared with ESD, UEMR generally offers shorter procedure times but lower R0 resection rates, especially for 20–30 mm lesions [7]. A propensity score analysis showed that ESD achieved superior R0 resection rates [7], although at the cost of substantially longer procedure times. Therefore, while ESD is preferable when complete R0 resection is mandatory in 20–30 mm lesions, UEMR may be a reasonable and minimally invasive option for smaller lesions (≤20 mm) without deep submucosal invasion or for non‐lifting lesions caused primarily by scarring. In addition, from an oncological standpoint, lesion size is generally correlated with the risk of deep submucosal invasion, and smaller lesions are generally less likely to harbor extensive invasion. This consideration further supported our decision to select UEMR in the present case. Rather than proceeding directly to ESD for all non‐lifting lesions, a strategy of selecting UEMR first—particularly for lesions where non‐lifting is due primarily to scarring, depending on location, size, and morphology—appears rational in terms of technical simplicity, shorter procedure time, and reduced risk of complications.

Regarding resection depth, specimens obtained by UEMR have been reported to contain a thinner submucosal layer than those obtained by CEMR (median ∼950 µm), but both generally achieve negative vertical margins [8]. Therefore, UEMR may provide sufficient vertical margins for microinvasive cancers with invasion <1000 µm. However, because UEMR specimens are thinner, uncertainty remains in securing negative margins when deep invasion is suspected.

Most reports of UEMR efficacy have focused on intermediate (10–20 mm) lesions or laterally spreading tumors larger than 20 mm, as well as on residual or recurrent lesions. Reports of small (≤10 mm) non‐lifting colorectal carcinomas, such as in this case, in which incomplete EMR was salvaged by en bloc curative UEMR, remain limited. Table 1 summarizes previous reports of UEMR for residual or recurrent colorectal lesions, including small (≤10 mm) and non‐lifting lesions. These studies support the feasibility of UEMR in scarred settings, while the present case is distinctive in that it clearly documents the non‐lifting sign with endoscopic images and demonstrates curative en bloc UEMR after failed CEMR, thereby providing additional educational value. However, further clarification of its role requires the accumulation of similar cases.

**TABLE 1:** Summary of reported cases of underwater endoscopic mucosal resection (UEMR) for residual or recurrent colorectal lesions, including small (≤10 mm) lesions.

First author, Year	Study design	Lesion type/size	N (UEMR)	Key outcomes	Clinical relevance/ Notes
Kim et al., 2014 [4]	Retrospective, single‐center	Recurrent adenomas at EMR scars (scarred, non‐lifting); mostly ∼10 mm; one invasive cancer included	36	En bloc 47%, complete 89%, recurrence 10%; no perforation, one delayed bleed	Demonstrated UEMR superiority over CEMR for scarred recurrences. Feasible, but oncological assurance is limited for invasive cases.
Ohmori et al., 2021 [9]	Retrospective, PSM	Residual/recurrent lesions after ER; median 8 mm (range 2–22)	30	En bloc 73% (ESD 100%), R0 41% (ESD 81%), no recurrence in either group; short procedure time, no AE	Highlighted UEMR as practical for small residual/recurrent lesions; however, ESD provided higher oncological assurance.
Takeuchi et al., 2022 [10]	Narrative review	Summarized outcomes of UEMR, including small (<10 mm) and recurrent lesions	−	Concluded that UEMR is feasible and safe for scarred small lesions	Provided an overall synthesis of existing evidence, serving as background to contextualize the present case.
Present case	Case report	Non‐lifting carcinoma after failed CEMR; 7 mm	1	En bloc, R0; well‐differentiated tubular adenocarcinoma; SM invasion 560 µm; negative margins	Illustrates that even a very small (<10 mm) non‐lifting carcinoma can be salvaged curatively with UEMR, emphasizing its role as a minimally invasive salvage option.

Abbreviations: AE, adverse event; CEMR, conventional EMR; EMR, endoscopic mucosal resection; ER, endoscopic resection; ESD, endoscopic submucosal dissection; SM, submucosal; UEMR, underwater endoscopic mucosal resection.

In conclusion, this case illustrates the potential role of UEMR for small non‐lifting carcinomas and highlights its educational value. Further accumulation of cases will be needed to confirm its utility.

## Author Contributions

Hiroshi Sawaguchi performed the endoscopic treatment and drafted the manuscript. Takuma Okamura supervised the endoscopic procedure. Hiroyoshi Ota contributed to the pathological analysis. Tadanobu Nagaya and Yugo Iwaya reviewed the clinical data. Takuma Okamura, Yugo Iwaya, Hiroyoshi Ota, and Tadanobu Nagaya critically revised the manuscript. All authors read and approved the final manuscript.

## Conflicts of Interest

The authors declare no conflicts of interest.
